# COVID-19-Associated Mental Health Impact on Menstrual Function Aspects: Dysmenorrhea and Premenstrual Syndrome, and Genitourinary Tract Health: A Cross Sectional Study among Jordanian Medical Students

**DOI:** 10.3390/ijerph19031439

**Published:** 2022-01-27

**Authors:** Iman Aolymat, Ashraf I. Khasawneh, Mohammad Al-Tamimi

**Affiliations:** Department of Basic Medical Sciences, Faculty of Medicine, The Hashemite University, P.O. Box 330127, Zarqa 13133, Jordan; ashrafkh@hu.edu.jo (A.I.K.); mohammad.altamimi@hu.edu.jo (M.A.-T.)

**Keywords:** COVID-19, dysmenorrhea, mental health, premenstrual syndrome (PMS), stress physiology, DASS-21

## Abstract

The physiology of reproduction is affected by psychological distress through neuroendocrine pathways. Historically, COVID-19 is one of the most stressful events with devastating consequences. This research aims to investigate the relationship between dysmenorrhea, PMS, and reproductive tract health on one hand, and COVID-19-related anxiety, depression, and stress on the other among medical students in Jordan. Medical students were invited through teaching platforms and social media to complete an online survey. SPSS software was used to analyze data. A total of 385 medical students participated in this research. Hence, 49.9% of the study population reported severe dysmenorrhea during COVID-19 compared to 36.9% before COVID-19 (*p* = 0.000). Dysmenorrhea was significantly associated with disruptions of sport and daily activities during COVID-19 (*p* = 0.015 and *p* = 0.002, respectively). The prevalence of PMS components, e.g., mastalgia, fatigue, headache, palpitation, and emotional and sleep disturbances, was raised during COVID-19 compared with before (*p* < 0.05). Symptoms of genitourinary tract infections, such as lower abdominal pain, vaginal discharge, genitalia rash/ulcers and itching, and urgency, were significantly increased after COVID-19 (*p* < 0.05). Positive Pearson correlations between COVID-19-associated mental health disorders and dysmenorrhea severity, PMS, and genital tract health abnormalities were observed (*p* < 0.05). The multiple linear regression model revealed that dysmenorrhea severity, PMS symptoms like palpitation, and genitourinary symptoms like lower abdominal pain and urgency were associated with worsening of depression, while dysuria was associated with a protective effect against depression. Moreover, it was observed that dysmenorrhea severity, PMS symptoms, such as headache and palpitation, and urinary urgency were associated with aggravation of anxiety. However, food craving and dysuria were protective against anxiety. Finally, dysmenorrhea severity, PMS symptoms of headache and palpitation, lower abdominal pain, and urgency were related to worsening of stress, whereas the premenstrual symptom of breast pain was a protective factor against stress. This work showed that COVID-19 pandemic-related psychological distress and menstrual, premenstrual, and genitourinary symptoms are closely related. Further future work is required to evaluate the long lasting-effects of the pandemic on mental health and the physiology of reproduction.

## 1. Introduction

Stress is a state of organic or psychological strain that disturbs normal physiology. Physiologically, the body responds to stressful stimuli by the stimulation of a group of interacting pathways, including the neuroendocrine systems. The hypothalamic–pituitary–adrenal (HPA) axis is one of the major pathways targeted by the stress response, leading to a high level of cortisol with consequent adverse effects on different body functions [1]. Furthermore, stress can disturb natural immunity by the activation of the HPA and sympathetic–adrenal–medullary (SAM) axis [1].

Menstrual cycle is a physiological process that encompasses several biological, psychological, and social components [2]. Painful menstruation or dysmenorrhea, which is caused by uterine contractions before or during menstruation, is one of the commonest menstrual disorders affecting females in their reproductive years, resulting in recurrent monthly impairments in quality of life. Dysmenorrhea negatively influences women’s daily routines, job or education attendance, sleeping, social life, and mental health [3]. Premenstrual syndrome (PMS) refers to behavioural, emotional, cognitive, and physiological disturbance during the luteal phase of the menstrual cycle ending with menstrual bleeding. It is caused by hormonal fluctuations during the menstrual cycle. PMS is associated with disruptions in daily activities, occupation, education, and social relationships [2,4,5]. Dysmenorrhea, PMS, and genital tract infections and psychological distress are closely related through stress-mediated modulation of the hypothalamic-pituitary-gonadal (HPG) axis [3,6,7,8,9,10].

About two years since the emergence of the global COVID-19 pandemic, one of the most stressful events historically caused by severe acute respiratory syndrome coronavirus 2 (SARS-CoV-2) [11] has appeared. The viral disease is associated with severe illnesses, such as pneumonia, acute respiratory distress syndrome (ARDS), multi-organ system failure, septic shock, and even death. Furthermore, some patients are affected by severe long-term health consequences following the illness, e.g., fatigue, respiratory, and neurological symptoms [11]. To contain the pandemic, several precautionary measures have been implemented worldwide, such as social distancing, self-isolation and quarantine, compulsory face masks, movement restrictions and total curfews, public services shutdown, including health services, online teaching, working from home, and compulsory vaccines. The pandemic-related health consequences and precautionary measures were associated with a considerable negative impact on the quality of life and mental health of the population. 

Recent reports showed that COVID-19 pandemic-related mental disorders were increased over the world [12,13]. Salari et al. reported that 29.6%, 31.9%, and 33.7% of the world population experienced stress, anxiety, and depression, respectively, during the COVID-19 pandemic [14]. The study also indicated that females and younger age groups are more vulnerable to mental health problems during the pandemic [14]. Moreover, various recent studies investigated the impact of the COVID-19 pandemic on the mental health of university students in particular. For example, a study carried out in Malaysia during the peak of COVID-19 pandemic and the lockdown reported that approximately 30% of university students suffered from different levels of anxiety [15]. A second study conducted a month later in Malaysia during the COVID-19-related online teaching found that more than half of university students showed moderate to severe anxiety [16]. Busetta et al. also investigated the anxiety levels among Italian university students during the period of COVID-19-related lockdown [17]. The study showed that high anxiety levels were reported by >50% of the university students, and >50% of the students started to experience anxiety, while >60% of the students who suffered from anxiety before the pandemic showed aggravated anxiety symptoms during the pandemic [17].

Even though some COVID-19-related restrictions were gradually eased by the governments, the mental health of university students is still negatively impacted. Ren et al. observed that when the universities were reopened after several months of closure due to COVID-19, 15.5% and 32.4% of Chinese college students experienced manifestations of anxiety and depression, respectively [18]. Moreover, Woon et al. reported that after the movement control order was lifted in Malaysia, and some strict social activities were resumed, 36.4%, 36.7%, and 42.4% of the university students experienced mild to extremely severe levels of depression, anxiety and stress, respectively [19]. The previous studies evaluating the impact of COVID-19 on the mental health of university students revealed that several factors, such as infected friends or relatives, quarantine, lockdown, financial problems, wearing masks, checking temperature routinely, female gender, online learning, and ambiguity of the future, are associated with a higher risk for developing mental disorders [15,16,17,18,19].

According to the literature, psychological distress and menstrual disturbances among university female students are closely associated. Rafique and Al-Sheikh [20] investigated the prevalence of dysmenorrhea and PMS, and their relationship with psychological problems among females studying health sciences. The study showed that approximately 40% of the female students were affected by high perceived stress, and more than 85% and 45% of the students showed symptoms of dysmenorrhea and PMS, respectively. The study has also shown that symptoms of dysmenorrhea and PMS are strongly correlated with high perceived stress [20]. Another study conducted among international female students in China illustrated that around a quarter of the international female students suffered from dysmenorrhea, and one third reported symptoms of PMS [21]. The study reported that the high stress of international relocation was positively associated with menstrual disorders [21].

A recent study by Phelan et al. investigated the consequences of COVID-19 pandemic on the reproductive health of females from the general population [22]. Similar to our study, changes in PMS due to the pandemic were examined. However, the impact of the pandemic on dysmenorrhea and genitourinary tract health was not investigated, and their work focused thoroughly on the disturbances in menstrual cycles characteristics (such as cycle length, days of bleeding, and amount of blood loss). Moreover, a validated stress questionnaire was not used to assess the impact of COVID-19 on the mental health of their study population compared to the previously validated depression, anxiety, and the depression, anxiety, and stress scale-21 questionnaire (DASS-21) [23] employed in our research. Additionally, the sample size of our research is representative, and we think that employing only 1031 women from different countries in the world is a relatively small sample size to generalize their study findings. Finally, to the best of our knowledge, our research is the first that provides general insight into the impact of COVID-19-related mental disorders on the reproduction health of females studying medicine.

Finally, females at their reproductive age represent a quarter of the world’s population [24], and dysmenorrhea, PMS, and genital tract disorders are common reproductive problems affected by mental status and impacting women’s quality of life and socioeconomic status. Therefore, this research aims to investigate the relationship between dysmenorrhea, PMS, and reproductive tract health on one hand, and COVID-19 -related anxiety, depression, and stress on the other among medical students in Jordan.

## 2. Materials and Methods

### 2.1. Sample and Data Collection

The online Raosoft sample size calculator [25] was used to obtain the estimated sample size based on a 95% confidence interval (CI), 50% response rate, and 5% margin of error. The estimated number of undergraduate medical students in Jordan is about 12,000 students, including males and females. The required sample size from both genders was 373, and the present work employed 385 female medical students from all 6 medical schools at Jordanian universities. This cross-sectional study was conducted during the early appearance of the COVID-19 pandemic in Jordan (after a 10-months duration). Only single medical undergraduate females aged 18 or older were involved in the study. An electronic survey was used to collect the data from participants through social media and teaching platforms. The questionnaire was initially developed in the English language. An independent, bilingual, native Arabic professional translator translated the questionnaire into Arabic (except for the Arabic version of the DASS-21 questionnaire, which was developed by Moussa et al. [26]). Section 1 of the questionnaire included an introduction containing study aims, and violation and confidentiality of participation. The other parts of the questionnaire encompassed multiple-choice questions, where participants could choose one answer among predefined answers (such as yes/no/I don’t know options) and/or rating questions using Likert scale style questions. Part 2 of the questionnaire included questions about demographic characteristics (such as age, height, weight, and age of menarche) and medical and gynecological history. Part 3 of the questionnaire included questions about the menstrual characteristics of study participants, which was submitted for publication elsewhere. Part 4 compromised questions about participants dysmenorrhea incidence, severity, impact on quality of life, and treatment options. The following part explored manifestations of PMS. Part 6 assessed genitourinary tract health of participants, including symptoms of gynecological or urinary tract infections (UTI). The last section consisted of the previously validated DASS-21 [23] to assess the influence of the COVID-19 pandemic on participants’ mental health. Arabic version of the DASS-21 questionnaire has been developed and validated previously. The Arabic version of DASS-21 had acceptable reliability, discriminated between depression, anxiety, and stress, but to a lesser extent than the English one, and showed good psychometric properties (Cronbach’s alpha = 0.883) [26,27,28]. DASS-21 has three sub-scales (Depression, Anxiety, Stress (DAS)). Each subscale has 7 questions. Each question is scored on a 4-point Likert scale, ranging from 0 (never) to 3 (most of the time). Scores of 10–13, 8–9, and 15–18 show mild depression, anxiety, and stress, respectively, while 14–20, 10–14, and 19–25 scores represent moderate depression, anxiety, and stress, respectively. Scores of 21–27, 15–19, and 26–33 correspond with severe depression, anxiety, and stress, respectively. Scores ≥28, ≥20, and ≥34 indicate extremely severe depression, anxiety, and stress, respectively [23]. Most of the responses during the early COVID-19 era in Jordan (10-months) were compared with 10-months before COVID-19 as a control.

### 2.2. Statistical Analysis

SPSS software (version 25, IBM Corporation, Armonk, NY, USA) was employed in data analysis. Data were shown as means ± SD for continuous variables or frequency (*N*) and percentages for categorical variables. The McNemar test and paired student *t*-test were used to compare categorical and continuous variables, respectively, 10 months before and after COVID-19. Pearson’s correlation coefficient (R) was used to identify the relationship between COVID-19-related mental health, and dysmenorrhea, PMS, or genitourinary tract problems. The associations between dysmenorrhea, PMS, and genitourinary symptoms (independent variables) with depression, anxiety, and stress scores after COVID-19 (dependent variables) were investigated using a multiple linear regression model. Confounding factors and multicollinearity were considered during the analysis. Continuous variables were mostly normally distributed according to Shapiro–Wilk test (*p* ˃ 0.05), histogram, and normal Q-Q plot. *p* value ≤ 0.05 was considered statistically significant.

### 2.3. Ethical Approval

The Hashemite University Institutional Review Board Committee reviewed and authorized this study. Moreover, informed electronic consent to participate in the research and publish the data was obtained from each student.

## 3. Results

### 3.1. Description of Study Participants

The study population included 385 female participants from all the medical schools in Jordan. All participants consented to participate in our online structured survey distributed to all female medical students. The demographic features and medical history of the girls in the 10 months before and after the COVID-19 pandemic appearance in Jordan are presented in Table 1. A significant increase in the mean body mass index (BMI), psychiatric disease frequency, and neurological disease was observed among participants during COVID-19 (*p* ≤ 0.001). The DASS-21 total score significantly increased from 22.68 before COVID-19 to 31.10 during COVID-19 (*p* = 0.000). Furthermore, during COVID-19, depression, anxiety, and stress subscore means were significantly increased among participants in comparison with before (*p* = 0.000) (Table 1).

### 3.2. COVID-19 Impact on Dysmenorrhea and Premenstrual Syndrome (PMS) of the Study Population 

Participants were requested to report the presence of dysmenorrhea, its severity, treatment, and effects on quality of life in addition to their PMS reported in the 10 months before and after the COVID-19 pandemic (Table 2). The incidence of dysmenorrhea during COVID-19 was higher (94.8%) than before (92.7%). Severe dysmenorrhea was reported more frequently and significantly in 49.9% of the study population during COVID-19 compared to 36.9% before COVID-19 (*p* = 0.000). Dysmenorrhea was significantly associated with disruptions of sport and daily activities during the pandemic (*p* = 0.015 and *p* = 0.002, respectively). Regarding the PMS changes, breast pain, sleep disturbances, palpitations, headache, fatigue, and emotional disturbances were significantly increased during the pandemic (*p* < 0.05, Table 2).

### 3.3. COVID-19 Impact on Genital Tract Health of Medical Students 

Participants were further requested to report symptoms of genital and UTI symptoms, the treatment modalities used to treat their symptoms, and their reaction to these health problems during COVID-19 (Table 3). The prevalence of lower abdominal pain, abnormal vaginal discharge, genital itching/rash/ulcer, and urgency were significantly increased during COVID-19 compared to before. More students were worried about their genital tract health during the COVID-19 pandemic compared to before (*p* = 0.009).

### 3.4. Correlations between COVID-19-Related Psychological-Distress and Dysmenorrhea, PMS or Genital Tract Health Using Pearson Correlations

Table 4 shows Pearson correlations between COVID-19-associated mental health disorders and dysmenorrhea, PMS, or genital tract health. The Pearson correlation test indicated a significant positive correlation (*p* < 0.05) between total DASS-21 score, and depression, anxiety, and stress subscores with dysmenorrhea severity after COVID-19. The total DASS-21 score and most DAS subscores were correlated positively with the interruption of academic education, family and social activities, sports, and routine daily activities during COVID-19 (*p* < 0.01, Table 4).

During the pandemic, the total DASS-21 score was positively correlated (*p* < 0.05) with all of the PMS components except food cravings. Likewise, depression and anxiety subscores were positively associated (*p* < 0.05) with all PMS components except for food craving during COVID-19. Finally, the stress subscore was positively related (*p* < 0.05) with all PMS components except for breast pain and food craving during COVID-19 (Table 4).

Positive correlations between total DASS-21 score and DAS subscores with lower abdominal pain, abnormal vaginal discharge, genital itching, urgency, and the status of being worried or embarrassed about genital tract health during COVID-19 (*p* < 0.01) were reported. Moreover, a positive link between the total DASS-21 score and the depression subscores with genital rash or ulcer during COVID-19 was observed (*p* < 0.05). The anxiety subscore was positively associated with groin lump during the pandemic (*p* < 0.01) (Table 4).

### 3.5. Associations between Dysmenorrhea, PMS and Urogenital Tract Health on One Hand and COVID-19-Associated Mental Health Disorders on the Other Hand Using Multiple Linear Regression

Table 5 shows the multiple linear regression model between dysmenorrhea, PMS, and genital tract health symptoms during COVID-19 on one hand and the level of COVID-19-associated depression symptoms on the other hand. The multiple linear regression analysis revealed that dysmenorrhea severity (B = 1.180, 95% CI = 0.352 to 2.321, *p* = 0.008), PMS symptoms such as palpitation (B = 12.364, 95% CI = 0.822 to 4.146, *p* = 0.004), genitourinary symptoms like lower abdominal pain (B = 2.470, 95% CI = 0.287 to 4.645, *p* = 0.027) and urgency (B = 2.318, 95% CI = 0.805 to 4.039, *p* = 0.003), and being embarrassed about genital tract problems (B = 3.214, 95% CI = 0.830 to 5.304, *p* = 0.007) were significantly associated with the worsening of COVID-19-associated depression. On the other hand, dysuria during the pandemic was associated with a protective effect against depression (B = −1.609, 95% CI = −3.257 to −0.102, *p* = 0.037).

Table 6 represents the multiple linear regression model between dysmenorrhea, PMS, and genital tract health symptoms during COVID-19 on one hand and the level of COVID-19-associated anxiety symptoms on the other hand. The multiple linear regression analysis revealed that dysmenorrhea severity (B = 0.966, 95% CI = 0.120 to 1.812, *p* = 0.025), PMS symptoms such as headache (B = 1.938, 95% CI = 0.347 to 3.529, *p* = 0.017), and palpitation (B = 3.735, 95% CI = 2.307 to 5.164, *p* = 0.000), urinary urgency (B = 3.078, 95% CI = 1.688 to 4.468, *p* = 0.000), and being embarrassed about genital tract problems (B = 2.178, 95% CI = 0.255 to 4.101, *p* = 0.027) were significantly associated with the aggravation of COVID-19-associated anxiety. In contrast, students who experienced food craving (B = −2.067, 95% CI = −3.587 to −0.547, *p* = 0.008) and dysuria symptoms (B = −1.365, 95% CI = −2.721 to −0.009, *p* = 0.048) showed a significant correlation with lower COVID-19-related anxiety symptoms.

Table 7 summarizes the multiple linear regression model between dysmenorrhea, PMS, and genital tract health symptoms during COVID-19 on one hand and the level of COVID-19-associated stress symptoms on the other hand. The multiple linear regression analysis revealed that dysmenorrhea severity (B = 1.185, 95% CI = 0.259 to 2.110, *p* = 0.012), PMS of headache (B = 1.979, 95% CI = 0.239 to 3.719, *p* = 0.026) and palpitation (B = 2.497, 95% CI = 0.935 to 4.059, *p* = 0.002), symptoms of lower abdominal pain (B = 2.055, 95% CI = 0.007 to 4.103, *p* = 0.049), and urgency (B = 2.626, 95% CI = 1.106 to 4.146, *p* = 0.001) were significantly related with the worsening of COVID-19-associated stress. However, the premenstrual symptom of breast pain was a protective factor against COVID-19-associated stress manifestations (B = −2.021, 95% CI = −3.576 to −0.466, *p* = 0.011).

## 4. Discussion

This study, to the best of our knowledge, is the first study evaluating the psychological effect of COVID-19 on medical students’ dysmenorrhea, PMS, and genital tract health during the early stages of the pandemic in Jordan. Our work indicates that dysmenorrhea severity and its negative impact on normal life activities was increased during the pandemic. The data also suggested that the prevalence of PMS and reproductive tract disorders was increased during COVID-19. These abnormalities were positively linked with a significant rise in COVID-19-linked psychological distress. On the other hand, dysmenorrhea, some PMS symptoms, and genitourinary abnormalities were associated with the exacerbation of COVID-19-related depression, anxiety, and stress symptoms.

Dysmenorrhea is one of the most prominent menstrual problems negatively affecting the quality of women’s life, and various reports in the literature described the prevalence of dysmenorrhea and its impact on women’s quality of life. A previous study conducted among female medical students in Jordan [29] showed that 55.8% of the students experienced moderate-severe dysmenorrhea, and 30.5% of the students missed their classes due to dysmenorrhea. The study also reported that dysmenorrhea was associated with significant disturbances in family and social relationships and sports activities [29]. This study showed a higher incidence of moderate-severe dysmenorrhea (82.4%) among the medical students in Jordan, and 43.1% of participants reported dysmenorrhea-related school absences before the pandemic. Furthermore, between two thirds and three quarters of the students showed interruptions in family and social interactions, sports, and daily routines. Additionally, several other studies [30,31,32] showed that dysmenorrhea and its negative impact on life quality is a major health issue affecting females across the world. 

To our knowledge, no previous report has investigated the association between infectious outbreak (other than COVID-19)-related psychological impact and dysmenorrhea severity, although various previous studies suggested a link between dysmenorrhea severity and mental health status of females [7,32]. Our work showed a positive link between dysmenorrhea severity and COVID-19-related mental disorders, which is consistent with previous studies identifying the relationship between stressful conditions and the severity of dysmenorrhea. 

Socioeconomic burdens of dysmenorrhea, such as demands for medical care and low productivity [33], require further efforts to identify the pathophysiological changes associated with dysmenorrhea to mitigate the negative impact of dysmenorrhea. It is well-established that the increased production of prostaglandins, such as PGF2a and PGF2, is the major cause of dysmenorrhea causing increased uterine contraction [33]. Changes in the structure and function of the central nervous system have also been described in psychological dysmenorrhea [33]. Similar functional mechanisms responsible for COVID-19-related dysmenorrhea might be present. However, this hypothesis needs further research that could help in the employment of therapeutic measures targeting the mechanism of pain, hence relieving the socioeconomic impacts of dysmenorrhea in similar situations.

Previous reports in the literature suggested a worsening of PMS symptoms during stressful events [8,9]. There were two studies which showed that females suffered more severe versions of PMS during the COVID-19 pandemic [22,34]. In line with these studies, our work showed that the prevalence of PMS symptoms, such as mastalgia, emotional disturbances, weakness, headache, palpitation, and sleep disturbances, was significantly increased during the pandemic. It is obvious that symptoms related to pain (such as headache and breast pain) and negative impact (such as emotional disturbances and sleep disorders) are the main symptoms with high prevalence during COVID-19, which could result in more debilitating PMS with a serious impact on female quality of life and mental status. 

The present study showed that dysmenorrhea severity is associated with higher levels of COVID-19-related depression, anxiety, and stress. However, the previous studies examining the association between dysmenorrhea and mental disorders showed inconsistent findings. Kabukçu et al. [35], Westling et al. [36], Zhao et al. [37], and Gagua et al. [38] have reported that dysmenorrhea severity is associated with a higher incidence of depression, anxiety, and stress disorders. Conversely, László et al. [39] showed no significant correlations between dysmenorrhea and higher incidence of psychological disorders, whereas Namvar et al. [40] reported that women with no dysmenorrhea exhibited higher anxiety symptoms than women with dysmenorrhea, and higher depression scores among women with dysmenorrhea than those without dysmenorrhea were reported.

In line with our findings, several previous researches in the literature suggested that menstrual abnormalities can worsen the mental status of affected female university students. A couple of studies have investigated the association between PMS and the psychological status of Iranian [41] and Saudi [42] female medical students. Both studies revealed that females with PMS exhibited more symptoms of depression, anxiety, and stress. Moreover, a couple of recent studies conducted among university female students also showed that increased PMS severity is associated with a worsening of depression, anxiety, and stress symptoms [43,44]. 

Stress controls the hypothalamic–pituitary–gonadal (HPG) axis resulting in the suppression of sex hormone release and stimulation of hypothalamic–sympathetic–neural pathways leading to norepinephrine secretion into the ovary [45]. Fluctuation in reproductive hormones during the menstrual cycle is the main suggested mechanism of PMS [45]. The COVID-19 pandemic, as with other identified stressors, could result in disturbances of the HPG axis and neuronal circuits [45], resulting in more severe symptoms of PMS. Moreover, COVID-19 infection survivors who participated in this study might have a direct effect on their reproductive system which express ACE2 receptors; the SARS-CoV-2 receptors [46]. Alternatively, infected students might have developed inflammatory reactions resulting in exacerbation of PMS similar to what was observed in other viral infections [47].

It is notable in this study that the COVID-19 pandemic was associated with increased frequency of genital and urinary tracts symptoms and signs. Additionally, positive correlations between genital and urinary tracts problems and COVID-19-related psychological distress were reported. These observations show parallelism with some other studies. Nansel et al. reported that bacterial vaginosis prevalence was increased among women with high levels of psychosocial stress [10]. Kissinger et al. found that lower reproductive tract infections in women were increased after stressful disastrous events such as hurricanes [48]. By contrast, two studies showed that the prevalence of genital tract infection during the COVID-19 pandemic was reduced or unchanged during COVID-19 among Jordanian [49] and Turkish women [50]. This contrast in results can be explained by variation in sociodemographic characteristics of the study participants. Additionally, both studies were conducted during the very early stages of the pandemic in Jordan and Turkey where COVID-19 morbidity and mortality was very low, which might have relieved the population stress about the pandemic. Furthermore, the religious and spiritual background of the studied population could have also resulted in these inconsistent findings, as multiple studies have shown the helpful role of spiritual and religious capital in coping with and protecting against COVID-19 psychological and mental stress [51,52]. Personality traits also play an important role in COVID-19-related changes and impacts where agreeable, extraverted, and conscientious persons had few COVID-19 related impacts compared to neurotic persons, who could be easily harmed [53,54,55]. 

Moreover, several previous studies described the relationship between psychological stress and the development of abnormal urinary tract signs and symptoms [56,57]. Although few recent reports in the literature showed that COVID-19 infection itself can cause symptoms of urinary tract infection [58,59,60], no study has investigated the association of COVID-19-related psychological distress with urinary tract health. Stress stimulates hypothalamic–pituitary–adrenal (HPA) and sympathetic–adrenal–medullary (SAM) axes. The activation of HPA and SAM axes is associated with the release of glucocorticoids and catecholamines, resulting in a modulated immune response and disturbances in vaginal sugar which are risk factors for genital and urinary tract infections [1]. This could be the pathophysiological mechanism for increased symptoms of genitourinary tract infections during the pandemic.

## 5. Conclusions

In summary, this research assessed the relationship between COVID-19-associated depression, anxiety, and stress and the high prevalence of severe dysmenorrhea, PMS, and genitourinary disorders among medical students. The findings of this study emphasize the importance of early recognition and intervention strategies to improve the physiological and psychological well-being of future medical practitioners.

## Figures and Tables

**Table 1:** Demographics, medical history, COVID-19 status and Depression, Anxiety and Stress Scale 21 (DASS-21) of the study population (*n* = 385).

Variable	Mean ± SD or N (%)		
Age (years)	19.89 ± 1.56		
Height (cm)	161.00 ± 7.78		
Menarche (age)	12.98 ± 1.286		
COVID-19 infection			
Yes	46 (11.9)		
No	251 (65.2)		
I don’t know	88 (22.9)		
	**Before COVID-19 Mean ± SD or N (%)**	**After COVID-19 Mean ± SD or N (%)**	***p* Value**
Weight (kg)	58.26 ± 11.27	59.14 ± 11.53	0.000 ***
BMI	22.87 ± 10.11	23.21 ± 10.76	0.001 **
Blood disease	31 (8.1)	38 (9.1)	0.118
Thyroid disease	8 (2.1)	10 (2.6)	0.625
Cancer	0 (0)	0 (0)	NA
Psychiatric disease	55 (14.3)	103 (26.8)	0.000 ***
Neurological diseases	13 (3.4)	30 (7.8)	0.000 ***
PCOS	37 (9.6)	38 (9.9)	1.00
Fibroid disease	2 (0.5)	2 (0.5)	NA
Uterine or ovarian surgery	0 (0)	0 (0)	NA
Chemotherapy or radiotherapy	2 (0.5)	0 (0)	NA
Hormonal therapy	23 (6)	23 (6)	1.00
DASS-21 total score	22.68 ± 16.80	31.10 ± 19.48	0.000 ***
Depression total score	7.38 ± 6.14	10.77 ± 7.22	0.000 ***
Anxiety total score	6.46 ± 5.75	8.76 ± 6.67	0.000 ***
Stress total score	8.83 ± 6.06	11.56 ± 6.81	0.000 ***

BMI; body mass index, PCOS; polycystic ovary syndrome, SD; standard deviation. ** *p* ≤ 0.01. *** *p* ≤ 0.001.

**Table 2:** Dysmenorrhea and premenstrual syndrome changes of female medical students before and after COVID-19.

Variable	Before COVID-19 Mean ± SD or N (%)	After COVID-19 Mean ± SD or N (%)	*p* Values
Dysmenorrhea	357 (92.7)	365 (94.8)	0.077
Dysmenorrhea severity			
Mild	40 (10.4)	37 (9.6)	0.250
Moderate	175 (45.5)	136 (35.3)	0.000 ***
Sever	142 (36.9)	192 (49.9)	0.000 ***
Dysmenorrhea treatment			
Herbal treatment	96 (24.9)	73 (19.0)	0.001 ***
NSAIDs	58 (15.1)	67 (17.4)	0.093
Paracetamol	111 (28.8)	133 (34.5)	0.003 **
Unknown treatment	27 (7.0)	32 (8.3)	0.267
Dysmenorrhea impacts			
Absenteeism from university teaching	166 (43.1)	154 (40.0)	0.213
Absenteeism from family and social activities	260 (67.5)	271 (70.4)	0.061
Absenteeism from sport	283 (73.5)	296 (76.9)	0.015 *
Interruption of daily activities	294 (76.4)	311 (80.8)	0.002 **
PMS symptoms			
Ovulation signs	314 (81.6)	320 (83.1)	0.238
Breast pain	267 (69.4)	283 (73.5)	0.000 ***
Emotional disturbances	361 (93.8)	369 (95.8)	0.039 *
Food craving	304 (79.0)	308 (80.0)	0.424
Fatigue	333 (86.5)	343 (89.1)	0.031 *
Acne	340 (88.3)	347 (90.1)	0.065
Headache	262 (68.1)	272 (70.6)	0.006 **
Palpitation	206 (53.5)	223 (57.9)	0.001 ***
Sleep disorder	276 (71.7)	294 (76.4)	0.001 ***
Swelling	299 (77.7)	300 (77.9)	1.00
Diarrhea or constipation	285 (74.0)	289 (75.1)	0.289

NSAIDs; nonsteroidal anti-inflammatory drugs, PMS; premenstrual syndrome. * *p* ≤ 0.05. ** *p* ≤ 0.01. *** *p* ≤ 0.001.

**Table 3:** Genital/urinary changes of medical students before and after COVID-19.

Variable	Before COVID-19 N (%)	After COVID-19 N (%)	*p* Values
Lower abdominal pain	323 (83.9)	334 (86.8)	0.007 **
Abnormal vaginal discharge	242 (62.9)	259 (67.3)	0.005 **
Genital rash or ulcer	88 (22.9)	103 (26.8)	0.003 **
Genital itching	219 (56.9)	249 (64.7)	0.000 ***
Groin lump	49 (12.7)	54 (14.0)	0.267
Dysuria	149 (38.7)	159 (41.3)	0.121
Urgency	172 (44.7)	192 (49.9)	0.000 ***
Genital/urinary tract symptoms treatment			
Never treated	154 (40.0)	155 (40.3)	1.00
Pharmacist/doctor phone consultation	5 (1.3)	6 (1.6)	0.219
Herbal treatments	36 (9.4)	35 (9.1)	1.00
Going to pharmacy	24 (6.2)	25 (6.5)	1.00
Going to private clinic	21 (5.5)	19 (4.9)	0.815
Going to MOH or RMS	10 (2.6)	10 (2.6)	1.00
Using home available drugs	35 (9.1)	38 (9.9)	0.648
Other treatment	5 (1.3)	8 (2.1)	0.375
Worried about genital tract health	65 (16.9)	81 (21.0)	0.009 **
Embarrassed about genital tract problems	61 (15.8)	62 (16.1)	1.00

MOH; Ministry of Health. RMS; Royal Medical Service. ** *p*≤ 0.01. *** *p*≤ 0.001.

**Table 4:** After COVID-19 Pearson correlation coefficients (r) for the relationship between dysmenorrhea, PMS, and genital/urinary symptoms with Depression, Anxiety and Stress Scale-21 (DASS-21) or subscales.

Variable	Depression r	Anxiety r	Stress r	DASS-21 r
Dysmenorrhea	−0.032	−0.056	−0.053	−0.049
Dysmenorrhea severity	0.193 **	0.238 **	0.229 **	0.233 **
Dysmenorrhea impacts				
Absenteeism from university teaching	0.235 **	0.261 **	0.221 **	0.254 **
Absenteeism from family and social activities	0.123 *	0.195 **	0.187 **	0.178 **
Absenteeism from sport	0.083	0.180 **	0.133 **	0.139 **
Interruption of daily activity	0.090	0.197 **	0.175 **	0.163 **
PMS				
Ovulation signs	0.164 **	0.181 **	0.177 **	0.185 **
Breast pain	0.111 *	0.167 **	0.085	0.128 *
Emotional	0.145 **	0.143 **	0.197 **	0.172 **
Food craving	0.009	−0.081	0.010	−0.021
Fatigue	0.161 **	0.168 **	0.197 **	0.186 **
Acne	0.113 *	0.118 *	0.148 **	0.134 **
Headache	0.285 **	0.354 **	0.323 **	0.340 **
Palpitation	0.329 **	0.448 **	0.349 **	0.398 **
Sleep disorder	0.216 **	0.271 **	0.238 **	0.257 **
Swelling	0.185 **	0.149 **	0.185 **	0.184 **
Diarrhea or constipation	0.279 **	0.280 **	0.271 **	0.294 **
Genital tract health				
Lower abdominal pain	0.270 **	0.253 **	0.261 **	0.278 **
Abnormal vaginal discharge	0.286 **	0.328 **	0.291 **	0.320 **
Genital rash or ulcer	0.142 **	0.078	0.089	0.110 *
Genital itching	0.254 **	0.214 **	0.185 **	0.232 **
Groin lump	0.074	0.142 **	0.053	0.095
Dysuria	0.054	0.073	0.090	0.077
Urgency	0.305 **	0.367 **	0.312 **	0.348 **
Worried about genital tract health	0.113 *	0.153 **	0.112 *	0.133 **
Embarrassed about genital tract problems	0.211 **	0.222 **	0.182 **	0.218 **

PMS; premenstrual syndrome. * Correlation is significant at the 0.05 level. ** Correlation is significant at the 0.01 level.

**Table 5:** Associations between dysmenorrhea, PMS, and genitourinary symptoms during COVID-19 with depression scores using multiple linear regression model.

Variable	B	95% CI	Standard Error	*p* Values
Dysmenorrhea	0.157	−2.776–3.330	1.552	0.859
Dysmenorrhea severity	1.180	0.352–2.321	0.501	0.008 **
Dysmenorrhea impacts				
Absenteeism from university teaching	1.516	−0.092–2.994	0.784	0.065
Absenteeism from family and social activities	−0.558	−2.553–1.817	1.111	0.741
Absenteeism from sport	−0.342	−2.905–2.109	1.275	0.755
Interruption of daily activity	−2.800	−5.466–0.295	1.465	0.078
PMS				
Ovulation signs	0.020	−1.895–1.957	0.979	0.975
Breast pain	−1.293	−2.924–0.386	0.841	0.132
Emotional	1.166	−2.402–5.234	1.941	0.466
Food craving	−0.186	−2.135–1.401	0.899	0.683
Fatigue	−0.278	−2.777–2.254	1.279	0.838
Acne	−0.736	−3.142–1.566	1.197	0.511
Headache	1.062	−0.665–3.038	0.941	0.208
Palpitation	2.364	0.822–4.146	0.845	0.004 **
Sleep disorder	−0.027	−1.862–1.945	0.968	0.966
Swelling	0.665	−1.068–2.493	0.905	0.432
Diarrhea or constipation	1.879	−0.018–3.655	0.934	0.052
Genital tract health				
Lower abdominal pain	2.470	0.287–4.645	1.108	0.027 *
Abnormal vaginal discharge	1.284	−0.607–2.855	0.880	0.202
Genital rash or ulcer	−0.255	−1.949–1.682	0.923	0.885
Genital itching	0.700	−0.835–2.550	0.861	0.320
Groin lump	−1.043	−3.259−1.016	1.087	0.303
Dysuria	−1.609	−3.257–−0.102	0.802	0.037 *
Urgency	2.318	0.805–4.039	0.822	0.003 **
Worried about genital tract health	−0.834	−2.991–1.117	1.044	0.370
Embarrassed about genital tract problems	3.214	0.830–5.304	1.137	0.007 **

PMS; premenstrual syndrome. * Correlation is significant at the 0.05 level. ** Correlation is significant at the 0.01 level.

**Table 6:** Associations between dysmenorrhea, PMS, and genitourinary symptoms during COVID-19 with anxiety scores using multiple linear regression model.

Variable	B	95% CI	Standard Error	*p* Values
Dysmenorrhea	0.305	−2.320–2.929	1.334	0.820
Dysmenorrhea severity	0.966	0.120–1.812	0.430	0.025 *
Dysmenorrhea impacts				
Absenteeism from university teaching	0.735	−0.591–2.061	0.674	0.277
Absenteeism from family and social activities	−0.470	−2.348–1.409	0.955	0.623
Absenteeism from sport	0.198	−1.957–2.352	1.096	0.857
Interruption of daily activity	−0.820	−0.591–1.656	1.259	0.515
PMS				
Ovulation signs	−0.051	−3.296–1.605	0.842	0.952
Breast pain	−0.743	−1.706–0.680	0.723	0.305
Emotional	0.444	−2.165–3.725	1.669	0.791
Food craving	−2.067	−2.838–−0.547	0.773	0.008 **
Fatigue	−0.814	−3.587–1.348	1.099	0.460
Acne	−0.397	−2.976–1.626	1.029	0.700
Headache	1.938	−2.420–3.529	0.809	0.017 *
Palpitation	3.735	0.347–5.164	0.726	0.000 **
Sleep disorder	0.594	2.307–2.230	0.832	0.476
Swelling	0.021	−1.042–1.552	0.778	0.978
Diarrhea or constipation	1.052	−1.509–2.631	0.802	0.191
Genital tract health				
Lower abdominal pain	1.574	−0.299–3.447	0.952	0.099
Abnormal vaginal discharge	1.417	−0.070–2.905	0.757	0.062
Genital rash or ulcer	−1.762	−3.322–−0.201	0.793	0.027 *
Genital itching	−0.470	−1.924–0.985	0.740	0.526
Groin lump	0.668	−1.169–2.505	0.934	0.475
Dysuria	−1.365	−2.721–−0.009	0.689	0.048 *
Urgency	3.078	1.688–4.468	0.707	0.000 **
Worried about genital tract health	0.292	−1.474–2.057	0.898	0.746
Embarrassed about genital tract problems	2.178	0.255–4.101	0.978	0.027 *

PMS; premenstrual syndrome. * Correlation is significant at the 0.05 level. ** Correlation is significant at the 0.01 level.

**Table 7:** Associations between dysmenorrhea, PMS, and genitourinary symptoms with stress scores using multiple linear regression model.

Variable	B	95% CI	Standard Error	*p* Values
Dysmenorrhea	0.212	−2.657–3.082	1.459	0.884
Dysmenorrhea severity	1.185	0.259–2.110	0.470	0.012 *
Dysmenorrhea impacts				
Absenteeism from university teaching	0.496	−0.954–1.946	0.737	0.502
Absenteeism from family and social activities	0.281	−1.773–2.335	1.044	0.788
Absenteeism from sport	−0.907	−3.263–1.449	1.198	0.449
Interruption of daily activity	−1.009	−3.716–1.698	1.377	0.464
PMS				
Ovulation signs	0.173	−1.637–1.984	0.921	0.851
Breast pain	−2.021	−3.576–−0.466	0.791	0.011 *
Emotional	2.480	−1.108–6.069	1.825	0.175
Food craving	−0.865	−2.527–0.797	0.845	0.307
Fatigue	0.281	−2.083–2.645	1.202	0.815
Acne	0.214	−1.998–2.427	1.125	0.849
Headache	1.979	0.239–3.719	0.885	0.026 *
Palpitation	2.497	0.935–4.059	0.794	0.002 **
Sleep disorder	0.296	−1.493–2.085	0.910	0.745
Swelling	0.586	−1.088–2.259	0.851	0.492
Diarrhea or constipation	0.919	−0.807–2.644	0.877	0.296
Genital tract health				
Lower abdominal pain	2.055	0.007–4.103	1.041	0.049 *
Abnormal vaginal discharge	1.260	−0.367–2.887	0.827	0.129
Genital rash or ulcer	−0.903	−2.609–0.804	0.868	0.299
Genital itching	−0.415	−2.006–1.176	0.809	0.608
Groin lump	−1.303	−3.312–0.706	1.021	0.203
Dysuria	−0.689	−2.172–0.793	0.754	0.361
Urgency	2.626	1.106–4.146	0.773	0.001 **
Worried about genital tract health	−0.190	−2.121–1.740	0.982	0.846
Embarrassed about genital tract problems	2.058	−0.044–4.161	1.069	0.055

PMS; premenstrual syndrome. * Correlation is significant at the 0.05 level. ** Correlation is significant at the 0.01 level.

## Data Availability

The data presented in this study are available in this article.
